# An Evaluation of Replacing Fishmeal with *Chlorella Sorokiniana* in the Diet of Pacific White Shrimp (*Litopenaeus Vannamei*): Growth, Body Color, and Flesh Quality

**DOI:** 10.1155/2022/8617265

**Published:** 2022-09-15

**Authors:** Menglu Li, Xiaoqin Li, Wenxiang Yao, Yuanyuan Wang, Xin Zhang, XiangJun Leng

**Affiliations:** ^1^National Demonstration Center for Experimental Fisheries Science Education, Shanghai Ocean University, Shanghai, China; ^2^Centre for Research on Environmental Ecology and Fish Nutrition (CREEFN) of the Ministry of Agriculture, Shanghai Ocean University, Shanghai, China; ^3^Shanghai Collaborative Innovation for Aquatic Animal Genetics and Breeding, Shanghai Ocean University, Shanghai, China

## Abstract

This study was conducted to investigate the effects of replacing fishmeal (FM) with *Chlorella sorokiniana* on growth and flesh quality of Pacific white shrimp, *Litopenaeus vannamei*. A control diet was formulated to contain 560 g/kg FM, and then chlorella meal was used to replace 0% (C-0), 20% (C-20), 40% (C-40), 60% (C-60), 80% (C-80), and 100% (C-100) of dietary FM, respectively. The six isoproteic and isolipidic diets were fed to shrimp (1.37 ± 0.02 g) for 8 weeks. The results showed that weight gain (WG) and protein retention (PR) of C-20 group were significantly higher than those of C-0 group (*P* < 0.05), while no significant differences were observed in WG and PR between C-0 and C-40 groups (*P* > 0.05). When the replaced level of FM by chlorella meal reached 60%, the WG of shrimp decreased and feed conversion ratio (FCR) increased significantly (*P* < 0.05). The quadratic regression analysis indicated that substituted fishmeal levels with chlorella meal were 20.50% and 28.25%, respectively, to obtain the highest WG and lowest FCR. In C-40 and C-60 groups, the body surface presented higher redness than the control (*P* < 0.05). No significant differences in the whole body and muscle composition, SOD, T-AOC, GSH-PX activities, MDA contents, total collagen content, steaming loss, texture property, free delicious amino acids contents, PUFAs, and n-3/n-6 PUFAs in flesh were observed among the three groups of C-0, C-20, and C-40 (*P* > 0.05). Compared to the control group, C-60, C-80, and C-100 groups showed lower flesh hardness, chewiness, shear force, and higher steaming loss and resilience (*P* < 0.05). There were no significant differences in serum TP, TG, GLU, and ALB contents, boiling loss, freezing loss, total free amino acids, SAFs and MUFAs among all the groups (*P* >0.05). Conclusively, in a diet containing 560 g/kg FM, chlorella meal could replace 40% dietary FM without negative effects on the growth and flesh quality, while increase the body redness of white shrimp.

## 1. Introduction

Pacific white shrimp, *Litopenaeus vannamei,* is the main cultured shrimp species in China and in the world. In 2020, the global cultural production of this shrimp reached 5.8 million tons [1]. With the rapid development of shrimp culture, the dietary demand for fishmeal is increasing. However, the fishmeal resource is limited, and the insufficient supply of fishmeal is becoming more and more serious [1], which makes it urgent to exploit suitable and sustainable protein sources to replace or decrease fishmeal inclusion.

Microalgae are rich in nutrients such as vitamins, minerals, proteins, polyunsaturated fatty acids, and carotenoids [2]. Microalgae are the natural diet for crustaceans and fish larvae, and they can release oxygen to regulate and stabilize water quality through photosynthesis. In addition, microalgae can form flocs with some abiotic components, which could promote the growth of shrimp [3, 4]. At present, the most widely used microalgae in aquaculture are chlorella. First, chlorella can be used as natural food for zooplankton such as rotifer, cladocera, and copepod, and rotifer fed with chlorella can be used as food for fish, shrimp, and shellfish, which can significantly improve the growth and development index of cultured animals [5]. Secondly, inoculating appropriate amount of chlorella into the culture system can optimize the population structure of plankton and improve water quality. At the same time, chlorella has the characteristics of acid resistance, antibiotic resistance, and higher thermal stability than general microbial preparations, so it is often used as animal feed additive, which can not only provide many aspects of nutrients for animals, but also enhance animal immunity. It contains rich protein (40%-60%), amino acids, polysaccharides, cytochrome, vitamins, minerals, unsaturated fatty acids, and chlorella growth factor (CGF). In addition to being used as feed additives, it can also be used as alternative protein sources for aquaculture animals. Previous studies have shown that replacing 47%-50% of dietary fishmeal with chlorella meal improved the growth and feed utilization of crucian carp (*Carassius carassius*) without adverse effects on liver histological structure [6]. In juvenile zebrafish (*Danio rerio*), the complete replacement of fishmeal with chlorella meal did not affect the growth and survival, but increased egg production and reduced blood lipid level [7]. In *L. vannamei*, the inclusion of chlorella meal reduced dietary soybean meal from 19.0% to 10.0%, and increased delicious amino acids content in flesh without affecting weight gain [8]. Also in *L. vannamei,* when 25% of dietary fishmeal was replaced by *Chlorella vulgaris* meal, the shrimp growth and the contents of long-chain polyunsaturated fatty acids as EPA, DHA, and ARA were increased, and the tolerance under hypoxia was also promoted [9].

To date, the application of chlorella meal to replace fishmeal in aquatic feed is still in the preliminary stage, and the effect of chlorella meal replacing fishmeal on flesh quality of *L. vannamei* has not been systematically studied. Therefore, this study was conducted to investigate the effect of replacing fishmeal with chlorella meal on the growth, body color, and flesh quality of *L. vannamei*, to explore the potential of chlorella meal as a substitute protein source for fishmeal in shrimp diet.

## 2. Materials and Methods

### 2.1. Ethical Statement

All animal care and use procedures were approved by the Institutional Animal Care and Use Committee of Shanghai Ocean University, and all authors clearly indicated that such guidelines have been followed.

### 2.2. Experimental Design and Diets

First, a control diet was designed with fishmeal as the main protein source (560 g/kg). Then, chlorella meal was used to replace 0% (C-0), 20% (C-20), 40% (C-40), 60% (C-60), 80% (C-80), and 100% (C-100) of dietary fishmeal by equalizing crude protein content to form six isoproteic (458.5 g/kg) and isolipidic (64.0 g/kg) diets. To balance the crude ash and phosphorus content of all diets, graded levels of bone meal was supplemented in fishmeal substituted diets. All ingredients were grounded and screened through 80-mesh sieve, then gradually mixed according to the formula (Table 1). After 15% water was added, the mixture was extruded to form sinking pellet (1.2 mm, pelleting temperature of 85 ± 5°C) with a single screw extruder (LX-75, Longxiang Food Machinery Factory, Hebei, China). The pellets were post-cooked in an oven at 95°C for 20 min, air-dried, and stored at 4°C until use.

*Chlorella sorokiniana* meal was provided by Demeter Bio-Tech Co., Ltd., Zhuhai, China. The contents of moisture, crude protein, crude lipid, crude ash, and carbohydrate in chlorella meal were 60.6 g/kg, 608.3 g/kg, 100.4g/kg, 54.1 g/kg, and 240.3 g/kg, respectively. Dietary amino acids, essential amino acid requirements of *L. vannamei* [10], and fatty acids compositions are shown in Tables 2 and 3.

### 2.3. Experimental Shrimp and Feeding Management

Pacific white shrimp larvae were purchased from a commercial farm in Shanghai, and temporarily reared in Binhai Aquaculture Station of Shanghai Ocean University (Shanghai, China). During the stocking period, shrimp were fed a commercial diet containing 40% crude protein and 6% crude lipid (Tongwei Feed Co., Ltd, Suzhou, China). After being deprived of diets for 24 h, 1200 healthy shrimp with initial body weight of (1.37 ± 0.02 g) were randomly assigned to 24 cages (1.0 m × 1.0 m × 1.2 m) locating in indoor cement pools (5.0 m × 3.0 m × 1.5 m) with 6 cages per pool. Thus, there were 6 treatments with 4 replicates (cages) per treatment and 50 shrimp per cage. During the feeding period, the daily feeding intake was about 4-8% of the body weight, and the shrimp were fed four times a day (7 : 00, 12 : 00, 17 : 00, and 23 : 00). According to the feed intake, water temperature, and weather conditions, the daily feed intake was appropriately adjusted to ensure no feed residue left within 2 h after feeding, and all cages were kept the similar feed intake. The feces and sediment at the bottom of the cages and pools were cleared by siphoning, and about 1/3 cultured water was renewed with filtered pond water every 4-7 days. During the feeding period, the dissolved oxygen, ammonia nitrogen, nitrite, temperature, pH, and salinity of water were ≥ 5.6 mg/L, ≤0.2 mg/L, ≤0.1 mg/L, 22-30°C, 7.8-8.5, and 0.5-1.0 ‰, respectively. The feeding trial lasted for 8 weeks.

### 2.4. Sampling

Before the feeding trial, twenty shrimp were randomly collected from the initial population and stored at −20°C for the analysis of initial proximate composition. At the end of the feeding trial, all shrimp were deprived of diets for 24 h, then counted and weighed for individual cage to calculate survival, weight gain (WG), and feed conversion ratio (FCR). Five shrimp from each cage were measured body weight and body length to calculate condition factor (CF), then haemolymph was syringed from the pericardial cavity and centrifuged at 4000 r/min for 10 min at 4°C. The supernatant was collected and stored at −80°C until use. Hepatopancreas were weighed to calculate hepatopancreas somatic index (HSI). The shrimp were peeled off shell, then the whole muscle was weighed to calculate meat yield [11], and preserved at −80°C for determining amino acids, fatty acids, biochemical indexes, and collagen content. Three shrimp per cage were used to determine the flesh texture with the second abdominal segments, and the third abdominal segments were steamed or cooked in boiling water for 5 min to measure steaming (boiling) loss. Meanwhile, the tail segments were stored at −20°C to determine the freezing loss. Another 3 shrimp per cage were boiled for 5 min to determine the color of body surface.

### 2.5. Measurement Indicators and Methods

#### 2.5.1. Growth Performance and Body Indices

Survival, weight gain (WG), feed conversion ratio (FCR), feed intake (FI), meat yield, hepatopancreas somatic index (HSI), and condition factor (CF) were calculated as follows:

(1) $$\begin{array}{c} \text{Survival }\left(\%\right)=100\times \left(\text{final shrimp number}/\text{initial shrimp number}\right),\\ \text{WG }\left(\%\right)=100\times \left[\left(\text{final weight }\left(\text{g}\right)-\text{initial weight }\left(\text{g}\right)\right)/\text{initial weight }\left(\text{g}\right)\right],\\ \text{FCR}=\text{feed intake }\left(\text{dry weight},\text{g}\right)/\left[\text{final weight }\left(\text{g}\right)-\text{initial weight }\left(\text{g}\right)\right],\\ \text{FI }\left(\%/\text{d}\right)=100\times \text{feed intake }\left(\text{dry weight},\text{g}\right)/\left[\left(\text{final weight }\left(\text{g}\right)+\text{initial weight }\left(\text{g}\right)\right)/2\times \text{days}\right].\\ \text{FI }\left(\text{g}/\text{shrimp}\right)=\text{feed intake }\left(\text{g}\right)/\text{final fish number}.\\ \text{Meat yield }\left(\%\right)=100\times \left[\text{weight of the abdominal and tail muscle }\left(\text{g}\right)/\text{final weight }\left(\text{g}\right)\right],\\ \text{HSI }\left(\%\right)=100\times \left[\text{final hepatopancreas weight }\left(\text{g}\right)/\text{final weight }\left(\text{g}\right)\right],\\ \text{CF }\left(\text{g}/\text{cm}3\right)=100\times \left[\text{final body weight }\left(\text{g}\right)/\text{body length }\left(\text{cm}\right)3\right]. \end{array}$$

#### 2.5.2. The Diet, Shrimp, and Muscle Proximate Composition and Nutrients Retention

The moisture, ash, crude lipid and crude protein contents in the diets, shrimp, and muscle were analyzed following the method of AOAC [12]. The moisture content was estimated by drying samples to constant weight at 105°C in a drying oven. After measuring the moisture content, the dried samples were ground into powder for the further determination. The crude protein content was determined using the Kjeldahl system method (2300 Auto analyzer, FOSS Tecator, Sweden). The crude lipid content was measured following the Soxhlet extraction method (FOSS Soxtec2050, Automatic Soxhlet extractor, Sweden). Ash content was analyzed by combusting samples in muffle furnace at 550°C for 6 h. Protein retention (PR) and lipid retention (LR) were calculated as follows:

(2) $$\begin{array}{c} \text{PR }\left(\%\right)=100\times \text{protein gain }\left(\text{g}\right)/\text{protein intake }\left(\text{g}\right),\\ \text{LR }\left(\%\right)=100\times \text{lipid gain }\left(\text{g}\right)/\text{lipid intake }\left(\text{g}\right). \end{array}$$

#### 2.5.3. The Amino Acid and Fatty Acid of Flesh and Diets

To determine the amino acid composition, 50 mg freeze-dried diets or 20 mg freeze-dried flesh was hydrolyzed with 6 M hydrochloric acid (HCl) for 24 h at 110°C in vacuum. Then, 0.5 mL of the hydrolysate was vacuum dried, diluted, filtered (0.22 *μ*m membrane filters), then used to determine the content of amino acids by Sykam 433D automatic amino acid analyzer (S-433D, Sykam, Germany).

Wet flesh sample (0.3 g) was homogenized with 9 mL of 5% trichloroacetic acid (TCA) for 1 min and ultrasonicated in ice water bath for 15 min, then centrifuged for 10 min (12,000 r/min, 4°C) after kept at 4°C for 2 h. The supernatant was collected and diluted to 10 mL after pH was adjusted to 2.0 ± 0.2 with 6 mol/L NaOH. One milliliter of extract was filtered using a 0.22 *μ*m membrane filter and applied to an automatic amino acid analyzer (Waters ACQUITY Ultra High Performance LC/MS, American) to determine the free amino acids composition in flesh.

The fatty acid composition of the diets and muscle was determined following the boron trifluoride method described by Yang et al. [13]. The extracted lipid from the diets or muscle was mixed with 2 mL of 14% boron trifluoride methanol solution. After 25 min of water bath at 100°C, benzene (2 mL) and methanol solution (2 mL) were added for another water bath (100°C, 25 min). Then, the samples were mixed with distilled water (2 mL) and n-hexane (2 mL) and centrifuged for 10 min (3000 r/min). The supernatant was mixed with n-hexane (0.5 mL). After centrifuging at 3000 r/min for 5 min, the supernatant was collected for fatty acid analysis with GC/MS (Agilent Technologies 7890B gas chromatograph-mass spectrometer, America).

#### 2.5.4. Hemolymph and Flesh Biochemical Indices

Serum glucose (GLU, Oxidase method), albumin(ALB, Bromocresol green method), total protein (TP, Coomassie brilliant blue method), triglyceride (TG, GPO-PAP method), total cholesterol (T-CHO, GPO-PAP method), malondialdehyde (MDA, TBA method), and lactic acid (LA, Colorimetric method) levels, total antioxidant capacity (T-AOC, ABST method), superoxide dismutase (SOD, WST-1 method), and glutathione peroxidase (GSH-Px, Colorimetric method) activities were measured using commercially available assay kits (Nanjing Jiancheng Bioengineering Institute, Nanjing, China).

The flesh hydroxyproline (Hyp) content was determined using hydroxyproline (Hyp) kits (Nanjing Jiancheng Bioengineering Institute, Nanjing, China) with alkaline hydrolysis method. The collagen content was calculated by multiplying the Hyp content by 8 [14]. The wet flesh samples (0.2 g) were homogenized with 4 times volume of Ringer's solution (consisting of 0.86% NaCl, 0.03% KCl, and 0.033% CaCl_2_) at 10000 r/min for 1 min, then the homogenates were heated for 70 min at 77°C and centrifuged at 12000 r/min for 30 min. The collagen content of the supernatant was the amount of heat-soluble collagen (HS).

Heat-insoluble collagen content (HIS) = Total collagen content—heat-soluble collagen content.

#### 2.5.5. Color Parameters

After cooking in boiling water for 5 min, the second abdominal segments of shrimp were measured for color analysis using the WSC-S colorimeter (an o/d light source, with a stability of Δ*Y* ≤ 0.6, Physical Optics Instrument Factory of Shanghai Precision Scientific Instrument Co., Ltd., Shanghai, China), including lightness (L∗), redness (a∗), and yellowness (b∗).

#### 2.5.6. Flesh Texture Characteristic and Water-Holding Capacity

After cooking for 5 min in boiling water, the flesh texture as hardness, chewiness, gumminess, cohesiveness, springiness, resilience, and shear force of the second abdominal segment without shell were determined with Universal TA Texture Analyzer (Shanghai Tengba Instrument Technology Co., Ltd., China). For flesh texture, the measuring conditions were as follows: a 25 mm × 25 mm cylindrical probe, contact sensing of 5 gf, test speed of 1 mm/s, and target mode of deformation (40% deformation and the time of 2 s). For flesh shear force, the second abdominal segment was placed perpendicularly, then Warner-Bratzler Shear cutter was used with contact sensing of 5 gf and test speed of 1 mm/s to record the maximum shear force.

After wiping off the surface water, the second abdominal segment without shell was weighed (W1), then steamed or cooked in boiling water for 5 min. The flesh was cooled to room temperature and weighed (W2) after the surface water was wiped off. For freezing loss measurement, a block of flesh (W1) was placed at −20°C for 24 h and then thawed at room temperature. The sample was weighed (W2) after the surface water was wiped off. The steaming, boiling, and freezing loss were calculated as follows:

(3) $$\begin{array}{c} \text{Steaming }\left(\text{boiling},\text{freezing}\right)\text{ loss }\left(\%\right)=100\times \left[\left(\text{W}1\left(\text{g}\right)–\text{W}2\left(\text{g}\right)\right)/\text{W}1\left(\text{g}\right)\right]. \end{array}$$

### 2.6. Statistical Analysis

The experimental data were presented as mean ± standard deviation. All data were analyzed using SPSS 26.0 statistical software. All evaluated variables were subjected to a one-way analysis of variance (ANOVA) to determine if there were significant (*P* < 0.05) differences between the observed responses. Tukey's multiple range tests were used to determine the statistical significance among groups. In addition, a follow-up trend analysis was performed using orthogonal polynomial contrasts to determine whether the significant effect was linear and/or quadratic.

## 3. Results

### 3.1. Growth Performance and Body Index

**Figure 1:**
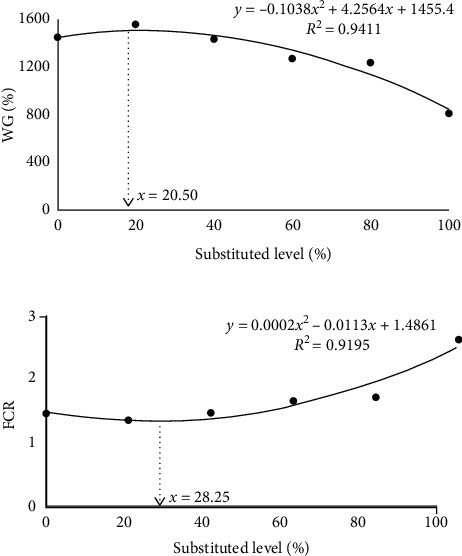
The quadratic regression analysis of the relationship between WG, FCR of *L. vannamei*, and dietary fish meal substituted level.

In Table 4, both linear and quadratic effects of FM replacement by chlorella meal were observed on WG, FCR and feed intake (%/d) (*P* < 0.05). Compared to the control, the WG of C-20 group was increased by 7.7% (*P* < 0.05), and FCR was decreased by 0.10 (*P* > 0.05), while no significant differences were observed in C-40 group (*P* > 0.05). When the replaced level of FM by chlorella meal reached 60%, the WG of shrimp was decreased, and FCR and feed intake (%/d) were increased significantly (*P* < 0.05). The CF of C-100 group and HSI of all substituted groups were significantly lower than those of the control (*P* < 0.05). Meat yield and feed intake of per shrimp showed no significant differences among all the groups (*P* > 0.05). The quadratic regression analysis indicated that substituted fishmeal levels with chlorella meal were 20.50% (YWG = −0.1038x2 + 4.2564x + 1455.4, R2 = 0.9411; Figure 1(a)) and 28.25%(YFCR  = 0.0002x2 − 0.0113x + 1.4861, R2 = 0.9195; Figure 1(b)), respectively, to obtain the maximum WG and minimum FCR.

### 3.2. Body Composition, Nutrients Retention, and Color Parameters

As shown in Table 5, linear and quadratic effects of dietary chlorella meal levels were observed on the contents of moisture, crude lipid, and the value of L∗ (*P* < 0.05). The crude ash content showed a positive linear relationship with dietary chlorella meal level (*P* < 0.05), but a negative linear relationship was found between crude protein content, b∗ value and dietary chlorella meal level (*P* < 0.05). When the substitution level of FM with chlorella meal increased, the values of L∗ and b∗ were significantly decreased, while a∗ had the highest level in C-40 and C-60 groups (*P* < 0.05). There were no significant differences in the contents of moisture, crude protein, crude lipid, crude ash, and b∗ among the three groups of C-0, C-20, and C-40 (*P* > 0.05). Compared to the control group, the crude protein content of C-100 group and the crude lipid content of C-60, C-80, and C-100 group were significantly decreased (*P* < 0.05). PR and LR were linearly and quadratically affected by dietary chlorella meal levels (*P* < 0.05), and C-20 group showed significantly higher values than those of the control group (*P* < 0.05). When the replaced level of FM by chlorella meal reached 60%, PR and LR were significantly decreased (*P* < 0.05).

### 3.3. Flesh Composition

As shown in Table 6, both linear and quadratic effects of dietary chlorella meal levels were observed on the contents of flesh moisture, crude lipid, and heat insoluble collagen (*P* < 0.05). There were no significant differences in the contents of flesh crude protein and total collagen among the three groups of C-0, C-20, and C-40 (*P* > 0.05). Compared to the control group, the contents of crude lipid of all substituted groups, crude protein of C-100 group, total collagen of C-60, C-80, and C-100 groups, and heat insoluble collagen of C-80 and C-100 groups were significantly decreased (*P* < 0.05).

### 3.4. Flesh Texture and Water-Holding Capacity

In Table 7, no significant differences in flesh hardness, chewiness, and shear force were observed among the three groups of C-0, C-20, and C-40 (*P* > 0.05), but the C-60, C-80, and C-100 groups showed significantly lower values than the control group except shear force in C-60 group (*P* < 0.05). A significantly negative linear trend was found between the increasing chlorella meal level and flesh gumminess (*P* < 0.05), and the gumminess of C-100 group was significantly lower than that of other groups (*P* < 0.05). Steaming loss was linearly and positively correlated with dietary chlorella meal (*P* < 0.05), and C-60, C-80, and C-100 groups showed significantly higher steaming loss than the control group (*P* < 0.05). There were no significant differences in flesh springiness, cohesiveness, boiling loss, and freezing loss among all the groups (*P* > 0.05).

### 3.5. Serum and Flesh Biochemical Indices

**Figure 2:**
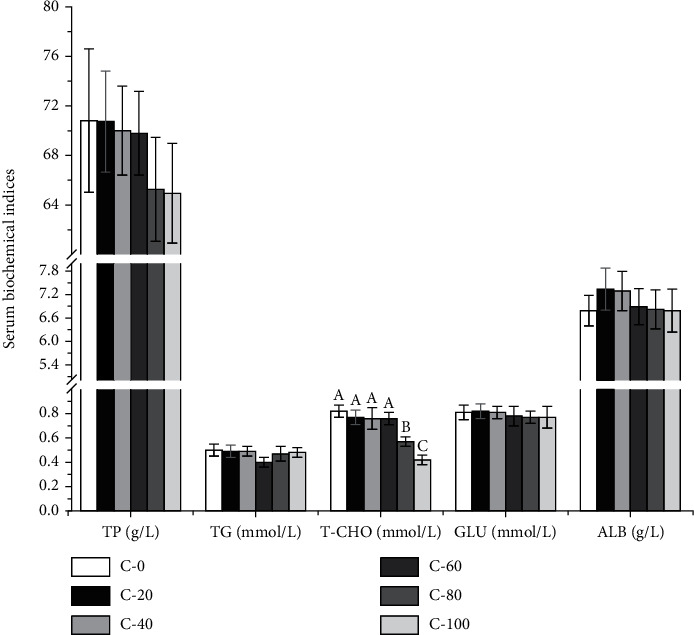
Effects of substituting FM with chlorella meal on serum biochemical indices of *L. vannamei*. Means in each bar with different superscript letters are significantly different (*P* < 0.05). TP: total protein; TG: triglyceride; T-CHO: total cholesterol; GLU: glucose; ALB: albumin.

**Figure 3:**
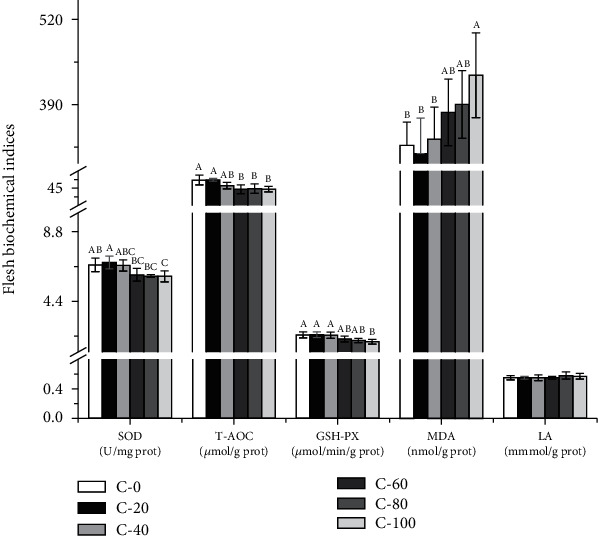
Effects of substituting FM with chlorella meal on flesh biochemical indices of *L. vannamei*. Means in each bar with different superscript letters are significantly different (*P* < 0.05). SOD: superoxide dismutase; T-AOC: total antioxidant capacity; GSH-PX: glutathione peroxidase; MDA: malondialdehyde; LA: lactic acid.

The serum and flesh biochemical indices of *L. vannamei* are shown in Figures 2 and 3. T-AOC activity in C-60, C-80, C-100 group, and GSH-PX activity in C-80 and C-100 group were significantly lower than those in the control group (*P* < 0.05). The C-100 group showed significantly higher MDA content and lower SOD activity than the control group (*P* < 0.05). The serum T-CHO contents were significantly decreased in C-80 and C-100 groups (*P* < 0.05). The replacement of FM with chlorella meal did not significantly affect the serum TP, TG, GLU, ALB, and flesh LA contents (*P* > 0.05).

### 3.6. Flesh Amino Acid Composition

In Table 8, when FM was completely replaced by chlorella meal (C-100 group), the contents of total amino acids (TAAs), essential amino acids (EAAs), tyrosine, and proline in flesh were significantly decreased (*P* < 0.05). In addition, the methionine content in all substituted groups and lysine content in C-80 and C-100 groups were significantly lower than those of the control group (*P* < 0.05).

A total of 17 free amino acids were detected in shrimp flesh, and arginine had the highest level, followed by proline and glycine. There were no significant differences in total free amino acids (TFAAs) in flesh among all the groups (*P* > 0.05). Significantly linear and quadratic relationships between glycine, delicious amino acids contents, and dietary chlorella meal level were detected (*P* < 0.05), and C-80 and C-100 group showed significantly lower contents than the control group (*P* < 0.05). The contents of lysine and histidine in C-60, C-80, and C-100 groups were also significantly lower than those in the control (*P* < 0.05) (Table 9).

### 3.7. Flesh Fatty Acid Composition

As shown in Table 10, PUFAs were the predominant class (59-60%), followed by SFAs (25-26%) and MUFAs (14%-15%). The ratio of n-3 PUFAs, n-6 PUFAs, and n-3/n-6 PUFAs showed no significant difference among the three groups of C-0, C-20, and C-40 (*P* > 0.05), but the ratio of n-3 PUFAs was decreased and the ratio of n-6 PUFAs was increased in C-60, C-80, and C-100 groups (*P* < 0.05). A negatively linear relationship was found between the ratio of EPA, DHA, and dietary chlorella meal, while LOA and ALA ratio showed positively linear relationship with dietary chlorella meal (*P* < 0.05). The substitution of FM by chlorella meal did not significantly affect the ratio of SFAs, MUFAs, ARA, and DPA in flesh (*P* > 0.05).

## 4. Discussion

### 4.1. Growth Performance

In a diet containing 400 g/kg fishmeal, the WG of Pacific white shrimp increased when 25% of diertary FM was replaced by chlorella meal [9]. In Nile tilapia (*Oreochromis niloticus*), chlorella meal was reported to replace 50% of dietary FM (222.3 g/kg) with positive impact on WG and SGR of fish [15]. When chlorella meal replaced 75% of dietary FM, the WG of African catfish (*Clarias gariepinus*) was increased, and FCR was decreased [16]. In the present study, the partial replacement of fishmeal (20%) with chlorella meal promoted the WG and PR of *L. vannamei*, which might result from the rich peptides, glycoproteins, polyamines, vitamins, phytohormones, and minerals, as well as special growth substance known as chlorella growth factor (CGF) [17]. The GCF can promote tissue regeneration and cell growth, stimulate the growth of beneficial microorganisms in the digestive tract to produce endogenous digestive enzymes, and promote protein assimilation [18]. In addition, the combination of appropriate amount of chlorella meal and fishmeal may produce complementary effects of proteins.

However, the growth performance of Pacific white shrimp was significantly decreased when the substituted level of dietary FM by chlorella meal reached 60%. In Atlantic cod (*Gadus morhua*), the increasing inclusion of *Nannochloropsis* sp. and *Isochrysis* sp. decreased the feed intake and growth performance, which may be due to the negative effect on the palatability of diet [19]. Generally, the carbohydrate content in chlorella is about 12-17% with the forms of starch, cellulose, and other polysaccharides [20]. It has been proved that some crustaceans such as Australian freshwater redclaw crayfish (*Cherax quadricarinatus*) [21] and giant freshwater prawn (*Macrobrachium rosenbergii*) [22] can secrete endogenous cellulase, but the ability to convert cellulose into simple sugars and use it is limited, so high fiber level will adversely affect the digestion and absorption of nutrients [23]. In the processing of chlorella meal, it could be considered to reduce the content of cellulose or supplement cellulase to degrade it.

Chlorella meal contains lower methionine and histidine levels than fishmeal, thus, the high replacement of dietary FM with chlorella meal decreased the methionine and histidine contents in diet. In Table 2, the contents of methionine and histidine were only 70.0% and 71.6% of the control group when FM was completely replaced by chlorella meal. Studies have suggested that an imbalanced amino acid profile of diet or amino acid deficiencies may result in growth retardation, poor feed intake, and feed utilization [24]. This is an important reason resulting in the decreased growth performance in low fishmeal and high chlorella meal diets. In the future, some limited amino acids as methionine and histidine could be supplemented to balance dietary amino acid composition in the application of chlorella meal.

Polyunsaturated fatty acids are essential nutrients for the growth of *L. vannamei* [25], and *L. vannamei* has dietary requirements for linoleic acid (LOA), alpha-linolenic acid (ALA), arachidonic acid (ARA), eicosapentaenoic acid (EPA), and docosahexaenoic acid (DHA) [26]. In this study, with the increasing proportion of dietary FM replaced by chlorella meal, the contents of EPA and DHA decreased, but the contents of ALA and LOA increased. Some studies had shown that shrimp have the ability to prolong and desaturate ALA to form polyunsaturated fatty acids such as EPA and DHA [27, 28]. However, this process consumes energy and the conversion capacity of shrimp is limited ([29]), and EPA were more biologically active and elicited significantly higher growth rates than PUFA [30, 31], which may be another important reason for the negative effects on shrimp growth when chlorella meal replaced high proportion of FM.

### 4.2. Color Parameters and Flesh Physical and Chemical Characteristics

Consumers believe that the color of aquatic animals is related to their nutritional value, health, freshness, and flavor. For aquatic products, especially shrimp, color is an important factor affecting consumers' evaluation of aquatic product price and purchase desire. In this study, the C-40 and C-60 group showed significantly higher redness (a∗) than the control group, while the yellowness (b∗) decreased in the high chlorella meal groups (Table 5). Microalgae are rich in carotenoid, and it has been proved that dietary microalgae improved the coloring effect of aquatic animals ([29, 32]). The major pigment in chlorella is chlorophyll (1-3% of dry algae cell weight), which has no coloring effect on aquatic animals, while carotenoids account for 0.4% of the dry matter basis [33], including astaxanthin (0.01 mg/g), canthaxanthin (0.5 mg/g), and lutein (4.6 mg/g) [34, 35]. Aquatic animals cannot synthesize astaxanthin by themselves and must obtain it from diets [36]. Although *L. vannamei* has the ability to convert zeaxanthin and lutein into astaxanthin, this process requires energy consumption with low efficiency [37]. In this study, the increase of chlorella meal inclusion increased the contents of zeaxanthin and lutein in diet, and shrimp might convert them into astaxanthin, leading to the increase of redness and yellowness of body surface. However, in the high chlorella meal and low fishmeal diets, the converted astaxanthin from lutein and zeaxanthin could not make up for the decrease of astaxanthin from fishmeal, resulting in the decrease of a∗ in C-80 and C-100 groups.

The moisture content and water-holding capacity of flesh directly affect the taste, mouth feel, flavour, color, tenderness, and the quality of flesh [38]. The water-holding capacity of flesh is usually evaluated by steaming, boiling, and freezing loss [39]. The loss of water would lead to the loss of water-soluble flavor compounds in flesh [40], therefore, lower water loss means stronger water-holding capacity and better flesh quality. In this study, the steaming loss of flesh increased when the substituted level of FM by chlorella meal reached 60%, which may be related to the decrease of antioxidant capacity and the increase of oxidative damage of flesh. As shown in Figure 3, when chlorella meal replaced high proportion of FM, the total antioxidant capacity of flesh decreased and the content of MDA increased. Similarly, when chlorella meal replaced 75% of FM in the diets of giant freshwater prawn [41] and African catfish [16], the activities of superoxide dismutase and catalase were also significantly decreased.

The texture characteristic of flesh is also an important factor in determining consumers' choice. Generally, the texture characteristics of flesh include hardness, shear force, springiness, chewiness, cohesiveness, and resilience, which could be measured by simulating the chewing movement of human's teeth. Compared with livestock and poultry flesh, aquatic products have higher muscle fiber density and smaller spacing between myofibrils [42]. The hardness directly determines the taste of flesh, and consumers prefer chewy shrimp [43]. In the present study, when the proportion of chlorella meal replacing FM reached 60%, the flesh hardness and shear force decreased significantly (Table 7), which may be related to the reduced content of muscle collagen. Collagen is the main protein in connective tissue, and its content, type, and structure affect the hardness, tenderness, and chewiness of flesh [44]. Especially, alkaline-insoluble collagen contains complete, mature, and cross-linked collagen molecules, contributing to the formation of elastic network and enhancing the tensile strength of muscle [45]. The relationship between collagen and hardness has been confirmed in Atlantic salmon (*Salmo salar* L.) [46], grass carp (*Ctenopharyngodon idellus*) [47], large yellow croaker (*Larimichthys crocea*) [48], Pacific white shrimp (*Litopenaeus vannamei* ) [49], farmed sea bass and wild sea bass (*Dicentrarchus labrax* L.) [50]. In this study, when the replaced level of FM by chlorella meal reached 60%, the total collagen content in flesh decreased significantly, and the heat insoluble collagen content in C-80 and C-100 groups also decreased significantly (Table 6), which may result from the absence of hydroxyproline in chlorella. When chlorella meal replaced high level of fishmeal, dietary hydroxyproline would be insufficient to support the normal synthesis of collagen.

In addition, flesh pH is also an important factor in evaluating the flesh quality, and the drastic changes in pH may lead to the degeneration of muscle [51]. After death, the aerobic pathway was interrupted and the muscle metabolism mode was changed to anaerobic status. Glycogen generated lactic acid through glycolytic reaction, decreasing the flesh pH, and causing protein denaturation [52]. In this study, there was no difference in the content of lactic acid among all the groups, which may be due to the low content of glycogen in shrimp muscle.

### 4.3. Flesh Nutrition and Flavor

As the most important indicators, the richness of protein and amino acids in flesh determines the nutritional value of flesh. In this study, the contents of methionine and histidine in diet decreased, while the contents of proline and alanine increased with the increase of substituted level of fishmeal by chlorella meal (Table 2). However, the contents of the four amino acids in flesh decreased in the high substituted groups, and the total amino acids and essential amino acids contents decreased significantly in C-100 group (Table 8), which was not completely consistent with dietary amino acid composition. The similar results were also reported in largemouth bass (*Micropterus salmoides*) [53] indicating that the amino acids composition in flesh is relatively stable to dietary amino acids composition.

Free amino acids in flesh significantly affect the flavor, and amino acid pyrolysis is one of the important chemical reactions to produce different flavors for meat product [54, 55]. Generally, the fresh sweetness of shrimp flesh comes from the free amino acids, especially the delicious amino acids as aspartic acid, glycine, glutamic acid, and alanine [56]. In this study, there were no differences in the content of total amino acids in shrimp flesh, but the contents of delicious amino acids and glycine decreased significantly in C-80 and C-100 groups (Table 9). In addition, free histidine presents bitter taste, and histamine will be produced from histidine after decarboxylation. High concentration of histamine will produce toxic effect [57]. In the present study, with the increase of the proportion of chlorella meal replacing FM, the content of free histidine in muscle decreased, consistent with the change of histidine content in diet (Table 2).

The composition of fatty acids also affects the flesh quality of aquatic products. On the one hand, the volatile compounds derived from the oxidation of unsaturated fatty acids give off aromatic smell for the fish, but on the other hand, lipid peroxidation could destroy the integrity of the biofilm and produce some aldehydes and alcohols with bad smell [58]. Studies have shown that the fatty acid composition of fish fed a single diet for a long time would be close to dietary fatty acid composition [59–62]. In this study, the n-6 PUFAs ratio including LOA in flesh increased with the increasing proportion of dietary chlorella meal, while the n-3 PUFAs ratio including DHA and EPA decreased (Table 10). For most animals, n-3 and n-6 PUFAs cannot be synthesized de novo, but can only be obtained from food or from precursors [63], thus, n-3 and n-6 PUFAs are significantly affected by the diet. Among PUFAs, EPA, and DHA have been proved various beneficial effects for human such as reducing cardiovascular diseases and regulating lipid metabolism [64]. However, Pakravan et al. [9] found that the contents of EPA, DHA, and ARA in diet decreased with the increasing amount of chlorella meal replacing FM, while the contents of ALA, EPA, and DHA in flesh of *L. vannamei* increased. In black tiger prawn (*Penaeus monodon*), the supplementation of 5% microalgae mixture (*Dunaliella* sp., *Chlorella* sp., and *Nannochloropsis* sp.) to diet did not affect the fatty acid composition of flesh [65]. The different results may be related to the microalgae species, inclusion level and diet formulation. In addition, the ratio of n-3/n-6 PUFAs may be more important than the content of n-3 PUFAs or n-6 PUFAs [66]. Glencross et al., [67] proposed that the ideal value of n-3/n-6 PUFAs is about 2.5 through modeling technology. In this study, the flesh n-3/n-6 PUFAs in the control group, C-20 and C-40 groups were about 2.4, but when chlorella meal replaced 60%, 80%, and 100% of fishmeal, the ratio decreased to 1.76, 1.61, and 1.00, respectively, indicating a decreased nutritional value of fatty acids.

## 5. Conclusion

In a basal diet with fishmeal inclusion of 560 g/kg, 20% fishmeal (112 g/kg) replacement with chlorella meal promoted the growth performance of *L. vannamei*. High replacement of fishmeal with chlorella meal decreased the growth performance, feed utilization, flesh hardness, chewiness, water-holding capacity, collagen content, free delicious amino acids, and the ratio of n-3/n-6 PUFAs. Based on the growth performance, body color and flesh quality, the replacement of fishmeal with chlorella meal was suggested to be 40% (224 g/kg).

## Figures and Tables

**Table 1:** Ingredients and proximate composition of experimental diets (air dry basis, g/kg).

Ingredients^1)^	C-0	C-20	C-40	C-60	C-80	C-100
Fish meal	560.0	448.0	336.0	224.0	112.0	0.0
Chlorella	0.0	135.1	270.4	405.6	540.9	676.2
Wheat flour	280.0	239.8	199.2	158.9	118.5	78.0
Fish slurry	50.0	50.0	50.0	50.0	50.0	50.0
Bone meal	0.0	19.3	38.7	58.0	77.3	96.6
Soybean oil	15.0	12.8	10.7	8.5	6.3	4.2
Soybean lecithin	15.0	15.0	15.0	15.0	15.0	15.0
Vitamin premix^2)^	5.0	5.0	5.0	5.0	5.0	5.0
Mineral premix^3)^	25.0	25.0	25.0	25.0	25.0	25.0
Microcrystalline cellulose	50.0	50.0	50.0	50.0	50.0	50.0
Total	1000.0	1000.0	1000.0	1000.0	1000.0	1000.0
Chemical composition						
Crude protein	458.5	456.3	458.7	457.0	459.0	459.5
Crude lipid	64.9	63.8	64.2	63.9	63.8	64.0
Crude ash	135.6	138.2	136.4	135.5	137.1	141.0
Dry matter	934.7	933.3	932.5	929.8	936.7	935.7
Carbohydrate^4)^	219.74	219.02	218.09	217.33	216.53	215.66
Total phosphorus^5)^	16.7	16.7	16.7	16.7	16.7	16.7

(1) The ingredients were purchased from the Yuehai Feed Company (Zhejiang, China), and the protein contents of ingredients are as follow: (1) TASA fish meal 682.1 g/kg, wheat flour 145.5 g/kg, and fish Slurry 393.8 g/kg. (2) One kilogram of vitamin premix contained: Vit A 10000000 IU, Vit D3 2500000 IU, Vit E 100000 IU, Vit K3 8600 mg, Vit B1 11760 mg, Vit B2 20000 mg, Vit B6 19600 mg, Vit B 1250 mg, D-biotin 200 mg, folic acid 3800 mg, niacinamide 59700 mg, d-pantothenic acid 44100 mg, and moisture ≤ 6%. (3) One kilogram of mineral premix contained: potassium 200.000 g, calcium 150.000 g, magnesium 58.200 g, cuprum 3.550g, zinc 5.400 g, manganese 1.950 g, cobalt 0.198 g, selenium 0.040 g, and iodine 0.248 g. (4) Carbohydrate content was calculated according to the carbohydrate content in ingredients. (5) Total phosphorus was calculated according to the phosphorus content in ingredients.

**Table 2:** Amino acid composition of experimental diets, fishmeal, chlorella meal, and essential amino acid requirements of *L. vannamei* (dry matter basis, g/kg).

Parameters	C-0	C-20	C-40	C-60	C-80	C-100	FM	Chlorella	*L. vannamei*
Essential amino acids (EAAs)									
Isoleucine	16.6	18.0	18.1	17.5	17.3	17.4	27.5	20.2	10.0
Leucine	34.4	35.7	36.0	36.0	37.5	38.2	52.6	43.4	17.0
Threonine	16.1	17.1	17.1	16.6	17.4	17.2	28.7	22.7	14.0
Phenylalanine	25.8	24.2	23.8	26.0	24.8	25.8	35.9	21.8	14.0
Histidine	16.9	13.7	12.9	12.1	12.1	12.1	20.7	7.7	8.0
Lysine	28.6	27.6	26.3	25.5	25.0	24.6	52.1	31.5	21.0
Arginine	26.5	24.5	25.1	27.3	28.9	25.6	40.9	28.5	25.0
Valine	20.5	21.3	21.9	21.9	22.3	22.4	33.7	25.5	14.0
Methionine	13.0	11.9	10.8	9.6	9.5	9.1	20.3	7.1	9.0
Non-essential amino acids (NEAAs)									
Aspartic acid	42.7	42.0	42.5	42.0	38.5	38.2	61.0	42.8	—
Serine	18.1	19.5	19.0	19.9	17.8	17.7	26.1	18.9	—
Glutamic acid	64.9	65.3	64.9	63.1	65.2	64.6	87.5	55.8	—
Tyrosine	14.9	13.9	16.3	16.1	14.9	16.0	22.9	17.1	—
Glycine	25.3	26.5	26.3	26.0	23.4	23.7	41.3	27.9	—
Alanine	29.4	31.6	31.2	31.1	31.9	31.8	44.2	42.5	—
Cysteine	2.4	2.4	2.5	2.4	2.1	2.5	4.1	2.5	—
Proline	20.2	21.3	20.7	20.6	20.5	21.0	28.4	32.5	—
Total amino acids (TAAs)	416.6	416.5	415.1	413.6	409.2	407.9	627.9	448.3	—

“—” means not determined.

**Table 3:** Fatty acids composition of experimental diets (percentage of total fatty acids, %).

Parameters	C-0	C-20	C-40	C-60	C-80	C-100
C14:0	2.11	2.21	2.27	2.12	2.16	2.08
C15:0	0.19	0.19	0.18	0.17	0.16	0.15
C16:0	12.35	13.66	14.17	17.13	17.98	18.84
C17:0	0.53	0.53	0.57	0.56	0.52	0.51
C18:0	3.19	3.20	3.22	3.28	3.13	3.34
C20:0	0.17	0.16	0.15	0.17	0.15	0.16
C24:0	0.18	0.19	0.18	0.18	0.17	0.18
SFAs	18.72	20.14	20.74	23.60	24.27	25.27
C16:1	4.00	4.06	4.44	4.70	5.34	5.43
C18:1	16.52	15.55	15.97	16.85	16.51	16.27
C20:1	0.63	0.58	0.61	0.63	0.60	0.58
C24:1	0.40	0.45	0.39	0.38	0.40	0.41
MUFAs	21.54	20.64	21.41	22.56	22.84	22.70
C18:2 LOA	24.46	25.28	27.32	28.00	32.26	35.85
C20:4 ARA	1.49	1.42	1.37	1.39	1.43	1.41
n-6 PUFAs	25.94	26.70	28.69	29.40	33.69	37.25
C18:3 ALA	4.99	5.60	6.54	8.12	8.93	9.13
C20:5 EPA	11.79	10.34	7.27	6.39	2.13	1.10
C22:5 DPA	1.91	1.76	1.79	1.71	1.70	1.70
C22:6 DHA	15.11	14.82	13.54	8.22	6.45	2.85
n-3 PUFAs	33.79	32.52	29.16	24.44	19.20	14.77

SFAs: saturated fatty acids; MUFAs: monounsaturated saturated fatty acids; PUFAs: polyunsaturated fatty acids; LOA:linoleic acid; ARA: arachidonic acid; ALA: linolenic acid; EPA: eicosapentaenoic acid; DPA: docosapentaenoic acid; DHA: docosahexaenoic acid.

**Table 4:** Effects of substituting FM with chlorella meal on growth performance and body index of *L. vannamei.*

Parameters	Diets						*Pr* > *F*		
	C-0	C-20	C-40	C-60	C-80	C-100	ANOVA	Linear	Quadratic
IBW (g)	1.37 ± 0.04	1.37 ± 0.03	1.37 ± 0.01	1.37 ± 0.09	1.37 ± 0.02	1.37 ± 0.05	—	—	—
FBW (g)	21.11 ± 0.48^b^	22.63 ± 0.07^a^	20.93 ± 0.79^b^	18.73 ± 0.30^c^	18.26 ± 0.56^c^	12.42 ± 0.32^d^	0.001	0.001	0.001
WG (%)	1440.5 ± 35.3^b^	1551.6 ± 5.1^a^	1427.4 ± 57.9^b^	1266.8 ± 22.2^c^	1232.5 ± 41.0^c^	806.6 ± 23.2^d^	0.001	0.001	0.001
Survival (%)	97.50 ± 2.51	99.50 ± 1.00	96.55 ± 1.91	99.02 ± 1.00	99.50 ± 1.00	99.50 ± 1.00	0.158	0.077	0.778
FI (%/d)	4.55 ± 0.11^a^	4.38 ± 0.23^a^	4.58 ± 0.17^a^	5.07 ± 0.06^b^	5.24 ± 0.14^b^	7.40 ± 0.17^c^	0.001	0.001	0.001
FI (g/shrimp)	28.62 ± 0.12	28.63 ± 0.07	28.58 ± 0.12	28.50 ± 0.08	28.77 ± 0.06	28.55 ± 0.08	0.107	0.316	0.039
FCR	1.45 ± 0.04^a^	1.35 ± 0.01^a^	1.46 ± 0.06^a^	1.64 ± 0.03^b^	1.70 ± 0.06^b^	2.59 ± 0.08^c^	0.001	0.001	0.001
CF (g/cm^3^)	1.12 ± 0.04^a^	1.12 ± 0.05^a^	1.11 ± 0.04^ab^	1.11 ± 0.03^ab^	1.09 ± 0.05^ab^	1.08 ± 0.03^b^	0.002	0.001	0.186
HSI (%)	4.35 ± 0.24^a^	3.83 ± 0.36^b^	3.63 ± 0.36^b^	3.87 ± 0.35^b^	3.90 ± 0.39^b^	3.95 ± 0.29^b^	0.001	0.051	0.001
Meat yield (%)	54.66 ± 1.44	56.46 ± 1.80	55.96 ± 1.88	55.92 ± 2.65	55.95 ± 2.41	55.22 ± 3.43	0.203	0.781	0.033

IBW: initial body weight; FI: feed intake; FBW: final body weight; WG: weight gain; FCR: feed conversion ratio; CF: condition factor; HSI: hepatopancreas somatic index. *Pr* > *F*: significant probability associated with the *F*-statistic. In the same row, values with different superscripts mean significant difference (*P* < 0.05), the same as the following tables.

**Table 5:** Effects of substituting FM with chlorella meal on body composition (g/kg), nutrients retention (%), and color parameters of *L. vannamei.*

Parameters	Diets						*Pr* > *F*		
	C-0	C-20	C-40	C-60	C-80	C-100	ANOVA	Linear	Quadratic
Moisture	735.2 ± 12.0^b^	733.8 ± 2.6^b^	728.4 ± 4.3^b^	739.6 ± 12.5^ab^	746.4 ± 10.6^ab^	755.4 ± 4.2^a^	0.005	0.001	0.021
Crude protein	204.0 ± 3.7^a^	201.1 ± 3.2^a^	205.1 ± 3.4^a^	197.3 ± 9.6^ab^	191.9 ± 8.9^ab^	185.3 ± 2.5^b^	0.006	0.001	0.097
Crude lipid	23.91 ± 0.92^a^	23.78 ± 1.68^a^	23.83 ± 1.23^a^	20.66 ± 1.79^b^	16.06 ± 0.95^c^	14.22 ± 0.48^c^	0.001	0.001	0.001
Crude ash	27.74 ± 2.34^b^	28.63 ± 0.47^ab^	28.84 ± 1.44^ab^	30.74 ± 0.72^ab^	31.18 ± 0.90^a^	30.95 ± 1.38^a^	0.007	0.001	0.459
Lightness (L∗)	61.29 ± 1.88^a^	56.80 ± 2.12^b^	56.74 ± 1.42^b^	56.69 ± 0.89^b^	56.72 ± 1.31^b^	56.56 ± 1.62^b^	0.001	0.001	0.001
Redness (a∗)	26.26 ± 1.70^c^	29.88 ± 2.29^ab^	30.71 ± 2.69^a^	30.74 ± 2.04^a^	28.11 ± 2.42^bc^	27.18 ± 2.75^c^	0.001	0.965	0.001
Yellowness (b∗)	38.20 ± 1.47^a^	37.69 ± 2.33^ab^	37.83 ± 2.65^ab^	35.93 ± 1.76^b^	35.89 ± 1.94^b^	36.91 ± 1.78^b^	0.001	0.001	0.679
PR	29.18 ± 0.76^b^	30.99 ± 0.12^a^	29.57 ± 0.63^b^	24.73 ± 0.17^c^	24.17 ± 0.15^c^	15.22 ± 0.43^d^	0.001	0.001	0.001
LR	24.70 ± 1.21^b^	27.72 ± 0.26^a^	24.63 ± 1.00^b^	18.23 ± 1.39^c^	16.10 ± 0.51^c^	8.54 ± 0.38^d^	0.001	0.001	0.001

PR: protein retention; LR: lipid retention.

**Table 6:** Effects of substituting FM with chlorella meal on flesh composition of *L. vannamei* (wet weight, g/kg).

Parameters	Diets						*Pr* > *F*		
	C-0	C-20	C-40	C-60	C-80	C-100	ANOVA	Linear	Quadratic
Moisture	734.6 ± 4.5^bc^	729.2 ± 5.5^c^	736.0 ± 5.3^bc^	739.2 ± 4.8^bc^	745.3 ± 4.6^ab^	753.3 ± 4.0^a^	0.001	0.001	0.009
Crude protein	238.1 ± 5.4^ab^	243.8 ± 5.5^a^	236.4 ± 6.0^ab^	228.8 ± 7.3^bc^	227.5 ± 4.0^bc^	220.0 ± 2.5^c^	0.002	0.001	0.168
Crude lipid	13.8 ± 0.5^a^	11.4 ± 0.9^b^	11.5 ± 0.4^b^	10.5 ± 0.8^b^	11.4 ± 0.8^b^	10.4 ± 1.0^b^	0.001	0.001	0.011
Crude ash	12.5 ± 0.3^b^	13.3 ± 0.1^ab^	13.3 ± 0.6^ab^	13.9 ± 0.1^a^	13.8 ± 0.8^a^	13.8 ± 0.3^a^	0.017	0.001	0.109
Total collagen	4.08 ± 0.30^a^	4.44 ± 0.20^a^	4.07 ± 0.33^a^	3.56 ± 0.27^b^	3.55 ± 0.18^b^	3.51 ± 0.22^b^	0.001	0.001	0.539
HS collagen	1.89 ± 0.12^ab^	1.82 ± 0.12^b^	1.90 ± 0.08^ab^	1.96 ± 0.16^ab^	2.02 ± 0.17^ab^	2.06 ± 0.14^a^	0.018	0.002	0.337
HIS collagen	2.19 ± 0.14^ab^	2.62 ± 0.15^a^	2.17 ± 0.20^ab^	1.60 ± 0.08^bc^	1.53 ± 0.07^c^	1.45 ± 0.07^c^	0.001	0.001	0.001

HS collagen: heat soluble collagen; HIS collagen: heat insoluble collagen.

**Table 7:** Effects of substituting FM with chlorella meal on flesh texture characteristics and water-holding capacity of *L. vannamei.*

Parameters	Diets						*Pr* > F		
	C-0	C-20	C-40	C-60	C-80	C-100	ANOVA	Linear	Quadratic
Hardness (gf)	541.8 ± 33.2^a^	505.0 ± 35.0^a^	506.3 ± 34.0^a^	440.0 ± 31.8^b^	446.0 ± 20.2^b^	362.8 ± 37.4^c^	0.001	0.001	0.048
Springiness	0.70 ± 0.016	0.71 ± 0.021	0.71 ± 0.019	0.71 ± 0.015	0.70 ± 0.017	0.72 ± 0.018	0.513	0.294	0.623
Chewiness (gf)	291.5 ± 17.7^a^	285.5 ± 16.0^a^	280.3 ± 18.1^a^	230.2 ± 18.9^b^	232.9 ± 21.5^b^	185.0 ± 17.6^c^	0.001	0.001	0.010
Gumminess (gf)	375.8 ± 24.1^a^	344.4 ± 27.2^a^	343.5 ± 44.9^a^	338.6 ± 29.1^a^	342.2 ± 35.5^a^	258.0 ± 24.4^b^	0.002	0.001	0.159
Cohesiveness (gf)	0.70 ± 0.03	0.71 ± 0.03	0.69 ± 0.01	0.70 ± 0.02	0.71 ± 0.01	0.71 ± 0.03	0.089	0.125	0.280
Resilience	0.58 ± 0.04^b^	0.58 ± 0.03^b^	0.57 ± 0.03^b^	0.58 ± 0.03^b^	0.61 ± 0.02^ab^	0.64 ± 0.04^a^	0.001	0.001	0.004
Shear force (gf)	2463.5 ± 205.7^a^	2420.3 ± 151.4^a^	2344.3 ± 143.1^a^	2328.3 ± 169.8^ab^	2107.3 ± 134.9^bc^	1974.8 ± 123.3^c^	0.001	0.001	0.049
Steaming loss (%)	18.92 ± 1.55^c^	20.63 ± 1.40^bc^	20.78 ± 2.16^bc^	23.55 ± 1.06^ab^	23.53 ± 1.70^ab^	25.32 ± 2.57^a^	0.001	0.001	0.979
Cooking loss (%)	21.77 ± 2.13	23.09 ± 2.39	23.11 ± 2.37	25.37 ± 2.58	24.68 ± 2.26	25.88 ± 2.49	0.068	0.003	0.723
Freezing loss (%)	2.24 ± 0.27	2.25 ± 0.13	2.32 ± 0.19	2.30 ± 0.20	2.33 ± 0.17	2.30 ± 0.28	1.000	0.829	0.882

**Table 8:** Effects of substituting FM with chlorella meal on flesh amino acid composition of *L. vannamei* (dry matter basis, g/kg).

Parameters	Diets						*Pr* > *F*		
	C-0	C-20	C-40	C-60	C-80	C-100	ANOVA	Linear	Quadratic
Essential amino acids (EAAs)									
Threonine	35.3 ± 1.5	36.5 ± 0.2	35.4 ± 2.4	35.2 ± 1.6	35.4 ± 1.2	35.0 ± 1.0	0.854	0.518	0.682
Phenylalanine	43.6 ± 1.9	47.9 ± 2.9	45.6 ± 2.7	45.7 ± 1.0	44.9 ± 4.0	41.8 ± 0.4	0.131	0.162	0.032
Lysine	72.8 ± 2.1^a^	71.5 ± 1.0^ab^	70.2 ± 1.4^ab^	69.9 ± 2.3^ab^	66.4 ± 3.3^bc^	64.1 ± 0.7^c^	0.001	0.001	0.221
Histidine	21.0 ± 0.6^ab^	21.7 ± 1.5^a^	20.4 ± 0.9^ab^	21.0 ± 0.8^ab^	21.1 ± 1.0^ab^	18.8 ± 0.6^b^	0.037	0.021	0.084
Arginine	80.9 ± 2.7	83.9 ± 0.7	84.7 ± 2.5	82.6 ± 2.4	82.5 ± 3.6	82.8 ± 0.6	0.500	0.799	0.197
Valine	42.2 ± 0.2	38.8 ± 3.6	39.5 ± 2.3	42.0 ± 2.0	40.0 ± 2.1	39.9 ± 2.0	0.399	0.640	0.632
Methionine	25.6 ± 0.6^a^	21.7 ± 1.2^b^	21.4 ± 1.1^b^	19.5 ± 0.9^bc^	16.9 ± 1.0^c^	17.1 ± 1.7^c^	0.001	0.001	0.088
Isoleucine	39.1 ± 0.5	37.4 ± 2.7	36.5 ± 2.5	34.8 ± 1.3	39.2 ± 0.7	36.7 ± 2.1	0.092	0.383	0.105
Leucine	65.2 ± 3.3	65.9 ± 0.1	66.2 ± 1.3	63.3 ± 2.5	64.2 ± 2.3	61.4 ± 0.6	0.098	0.016	0.193
Non-essential amino acids (NEAAs)									
Tyrosine	35.9 ± 0.5^a^	35.0 ± 0.5^a^	34.2 ± 1.1^a^	34.5 ± 0.5^a^	33.9 ± 2.1^ab^	31.3 ± 0.2^b^	0.004	0.001	0.182
Aspartic acid	82.7 ± 1.9	83.2 ± 2.3	84.4 ± 2.0	82.8 ± 3.2	83.1 ± 2.6	81.4 ± 2.8	0.796	0.504	0.285
Proline	54.8 ± 4.2^a^	54.6 ± 0.1^a^	51.7 ± 5.5^ab^	49.3 ± 1.9^ab^	46.0 ± 0.4^ab^	43.7 ± 4.5^b^	0.010	0.001	0.530
Serine	35.4 ± 1.2	35.5 ± 0.4	34.5 ± 2.7	35.7 ± 1.9	36.2 ± 1.0	34.7 ± 0.5	0.750	0.957	0.825
Glutamic acid	127.8 ± 2.2	136.3 ± 1.1	135.7 ± 2.8	135.4 ± .58	132.1 ± 1.0	127.8 ± 5.0	0.028	0.454	0.002
Glycine	55.0 ± 1.6	52.5 ± 2.0	54.6 ± 0.7	54.0 ± 2.3	56.4 ± 3.9	53.9 ± 4.5	0.672	0.672	0.939
Alanine	54.8 ± 1.2	54.7 ± 0.3	54.6 ± 2.5	52.6 ± 2.4	53.5 ± 1.3	53.7 ± 1.7	0.628	0.230	0.551
Cysteine	6.0 ± 0.4^a^	5.5 ± 0.8^a^	5.1 ± 0.5^ab^	4.2 ± 0.4^bc^	3.1 ± 0.1^c^	3.1 ± 0.1^c^	0.001	0.001	0.666
Total essential amino acids (TEAAs)	425.7 ± 11.4^a^	425.2 ± 5.8^a^	4200.±10.1^ab^	414.0 ± 3.4^ab^	410.6 ± 10.5^ab^	397.5 ± 7.0^b^	0.013	0.001	0.242
Total amino acids (TTAs)	877.9 ± 13.1^a^	882.4 ± 6.4^a^	874.8 ± 20.8^a^	862.3 ± 17.2^ab^	8552.±8.6^ab^	827.2 ± 25.2^b^	0.015	0.001	0.092

**Table 9:** Effects of substituting FM with chlorella meal on flesh free amino acid composition of *L. vannamei* (wet weight, mg/100 g).

Parameters	Diets						*Pr* > *F*		
	C-0	C-20	C-40	C-60	C-80	C-100	ANOVA	Linear	Quadratic
Aspartic acid ∗	3.51 ± 0.10	3.23 ± 0.30	3.47 ± 0.30	3.21 ± 0.19	3.30 ± 0.08	3.46 ± 0.26	0.439	0.794	0.206
Threonine	147.4 ± 0.54	149.1 ± 5.30	148.2 ± 11.98	149.6 ± 9.12	146.8 ± 3.46	147.1 ± 5.78	0.995	0.847	0.701
Serine	18.02 ± 0.20	18.10 ± 0.49	18.33 ± 0.94	18.15 ± 1.15	18.10 ± 0.62	18.14 ± 0.44	0.996	0.912	0.731
Glutamic acid∗	44.83 ± 4.53	45.39 ± 3.86	46.21 ± 2.41	45.59 ± 3.01	44.65 ± 1.62	46.43 ± 2.77	0.975	0.742	0.955
Glycine∗	647.5 ± 38.0^a^	652.1 ± 37.7^a^	638.8 ± 26.2^ab^	638.7 ± 16.9^ab^	561.6 ± 31.8^bc^	525.0 ± 18.8^c^	0.001	0.001	0.012
Alanine∗	248.2 ± 12.94	242.3 ± 6.13	250.6 ± 4.03	240.2 ± 10.32	244.0 ± 10.08	241.3 ± 8.85	0.704	0.392	0.966
Cysteine	6.63 ± 0.34	6.60 ± 0.37	6.20 ± 0.16	6.23 ± 0.22	6.25 ± 0.10	6.51 ± 0.36	0.255	0.245	0.054
Valine	24.82 ± 0.77	24.79 ± 2.21	24.96 ± 1.28	23.81 ± 0.81	23.88 ± 1.76	23.73 ± 1.59	0.804	0.221	0.898
Methionine	6.83 ± 0.20	6.87 ± 0.34	6.79 ± 0.47	6.31 ± 0.30	6.31 ± 0.38	6.03 ± 0.41	0.061	0.004	0.525
Isoleucine	7.92 ± 0.15	7.87 ± 0.26	7.86 ± 0.21	7.94 ± 0.23	7.66 ± 0.18	7.89 ± 0.31	0.693	0.528	0.798
Leucine	20.62 ± 0.38	20.81 ± 1.22	21.05 ± 0.79	20.15 ± 0.85	20.18 ± 0.83	20.28 ± 1.31	0.797	0.344	0.797
Threonine	17.45 ± 1.02	17.59 ± 0.40	17.54 ± 0.82	17.30 ± 1.23	17.87 ± 0.73	17.36 ± 1.32	0.982	0.974	0.882
Phenylalanine	11.26 ± 0.70	12.00 ± 0.92	11.49 ± 0.55	11.10 ± 0.63	11.53 ± 1.08	11.28 ± 0.64	0.769	0.648	0.785
Lysine	110.4 ± 1.99^a^	109.4 ± 5.07^a^	101.3 ± 3.84^ab^	92.30 ± 3.78^bc^	88.95 ± 3.61^c^	87.63 ± 6.40^c^	0.001	0.001	0.460
Histidine	25.34 ± 1.42^a^	24.45 ± 0.84^a^	23.32 ± 0.98^a^	18.04 ± 0.74^b^	16.16 ± 0.85^bc^	14.96 ± 0.73^c^	0.001	0.001	0.385
Arginine	779.6 ± 32.19	786.1 ± 61.16	798.4 ± 29.32	813.2 ± 73.91	807.6 ± 27.83	813.6 ± 71.99	0.948	0.352	0.797
Proline	669.7 ± 35.17	654.7 ± 27.79	668.7 ± 35.81	660.3 ± 23.55	650.2 ± 21.15	656.2 ± 12.60	0.932	0.510	0.954
Delicious amino acids (DAAs)	944.0 ± 43.54^a^	943.0 ± 31.88^a^	939.0 ± 25.64^a^	927.7 ± 5.18^ab^	853.5 ± 25.87^bc^	816.2 ± 12.85^c^	0.001	0.001	0.007
Total free amino acids (TFAAs)	2790.0 ± 40.13	2781.2 ± 106.8	2782.8 ± 16.17	2782.5 ± 105.0	2675.1 ± 32.18	2646.9 ± 70.07	0.072	0.007	0.163

DAAs: delicious amino acids (∗).

**Table 10:** Effects of substituting FM with chlorella meal on flesh fatty acid composition of *L. vannamei* (percentage of total fatty acids, %).

Parameters	Diets						*Pr* > *F*		
	C-0	C-20	C-40	C-60	C-80	C-100	ANOVA	Linear	Quadratic
C14:0	0.36 ± 0.012	0.35 ± 0.006	0.35 ± 0.010	0.35 ± 0.006	0.35 ± 0.006	0.35 ± 0.010	0.765	0.812	0.664
C15:0	0.22 ± 0.006	0.22 ± 0.011	0.22 ± 0.006	0.23 ± 0.025	0.22 ± 0.006	0.23 ± 0.015	0.948	1.000	0.891
C16:0	15.73 ± 0.27	15.27 ± 0.42	15.33 ± 0.44	15.48 ± 0.57	15.31 ± 0.47	15.21 ± 0.32	0.700	0.281	0.698
C17:0	0.73 ± 0.015	0.73 ± 0.015	0.73 ± 0.006	0.74 ± 0.023	0.73 ± 0.038	0.72 ± 0.015	0.923	0.703	0.438
C18:0	8.77 ± 0.14	8.73 ± 0.07	8.79 ± 0.17	8.78 ± 0.06	8.76 ± 0.13	8.71 ± 0.20	0.974	0.733	0.695
C20:0	0.47 ± 0.046	0.44 ± 0.006	0.44 ± 0.015	0.45 ± 0.021	0.47 ± 0.020	0.45 ± 0.025	0.447	0.852	0.443
SFAs	26.29 ± 0.37	25.74 ± 0.47	25.86 ± 0.53	26.03 ± 0.55	25.85 ± 0.45	25.66 ± 0.56	0.683	0.290	0.832
C16:1	1.11 ± 0.04	1.11 ± 0.03	1.05 ± 0.04	1.12 ± 0.06	1.04 ± 0.02	1.10 ± 0.05	0.123	0.432	0.377
C18:1	12.75 ± 0.18	12.69 ± 0.15	12.76 ± 0.18	12.65 ± 0.05	12.44 ± 0.20	12.56 ± 0.23	0.379	0.067	0.700
C20:1	0.56 ± 0.006^a^	0.58 ± 0.012^a^	0.59 ± 0.012^a^	0.51 ± 0.01^b^	0.45 ± 0.01^c^	0.37 ± 0.01^d^	0.001	0.001	0.001
MUFAs	14.42 ± 0.19	14.38 ± 0.17	14.41 ± 0.21	14.29 ± 0.03	14.00 ± 0.14	14.03 ± 0.20	0.024	0.002	0.307
C18:2 LOA	12.85 ± 0.57^d^	12.88 ± 0.10^d^	13.00 ± 0.27^d^	16.87 ± 0.47^c^	18.41 ± 0.65^b^	25.64 ± 0.28^a^	0.001	0.001	0.001
C20:2	2.14 ± 0.02	2.12 ± 0.04	2.12 ± 0.03	2.12 ± 0.05	2.09 ± 0.04	2.13 ± 0.06	0.730	0.426	0.360
C20:4 ARA	2.63 ± 0.14	2.72 ± 0.03	2.59 ± 0.17	2.64 ± 0.22	2.58 ± 0.27	2.40 ± 0.11	0.404	0.101	0.265
n-6 PUFAs	17.62 ± 0.68^d^	17.73 ± 0.13^d^	17.70 ± 0.39^d^	21.63 ± 0.61^c^	23.02 ± 0.34^b^	30.17 ± 0.18^a^	0.001	0.001	0.001
C18:3 ALA	0.77 ± 0.06^d^	1.29 ± 0.05^d^	1.33 ± 0.07^d^	3.09 ± 0.03^c^	5.09 ± 0.02^b^	8.95 ± 0.75^a^	0.001	0.001	0.001
C20:5 EPA	19.70 ± 0.17^b^	20.70 ± 0.27^a^	20.67 ± 0.63^ab^	18.51 ± 0.43^c^	17.95 ± 0.25^c^	11.63 ± 0.23^d^	0.001	0.001	0.001
C22:5 DPA	0.84 ± 0.021	0.83 ± 0.006	0.81 ± 0.006	0.81 ± 0.006	0.81 ± 0.015	0.83 ± 0.010	0.042	0.111	0.007
C22:6 DHA	20.37 ± 0.63^a^	19.32 ± 0.64^a^	19.20 ± 0.92^a^	15.63 ± 0.47^b^	13.29 ± 0.30^c^	8.73 ± 0.33^d^	0.001	0.001	0.001
n-3 PUFAs	41.67 ± 0.54^a^	42.16 ± 0.76^a^	42.02 ± 0.79^a^	38.04 ± 0.16^b^	37.14 ± 0.22^b^	30.14 ± 0.89^c^	0.001	0.001	0.001
PUFAs	59.29 ± 0.19	59.88 ± 0.63	59.72 ± 0.47	59.68 ± 0.57	60.16 ± 0.40	60.31 ± 0.75	0.295	0.042	0.893
n-3/n-6 PUFAs	2.37 ± 0.12^a^	2.38 ± 0.06^a^	2.38 ± 0.10^a^	1.76 ± 0.05^b^	1.61 ± 0.03^b^	1.00 ± 0.03^c^	0.001	0.001	0.001

SFAs: saturated fatty acids; MUFAs: monounsaturated fatty acids; PUFAs: polyunsaturated fatty acids.

## Data Availability

All data generated or analyzed during this study are included in this article.
